# Supplementary material for: Are carbon emissions trading and green financial instruments synergistic? -Comprehensive quantitative research based on content analysis
Source: PLoS One. 2024 Mar 7;19(3):e0298601. doi: 10.1371/journal.pone.0298601 (PMC10919601; doi:10.1371/journal.pone.0298601)
Supplement: S1 Data — (ZIP) [file pone.0298601.s001.zip › Data/地级市Z_F工作报告.zip!/┴╔─■/╔≥╤⌠╩╨/└┤╫╘╣1⁄2╓┌║┼í╛┬φ┐╦╩2╛▌═°í┐_2010.docx]

　　现在，我代表市人民政府向大会报告工作，请予审议，并请市政协委员提出意见和建议。

　　一、2009年政府工作回顾

　　2009年，是新世纪以来我市经济发展最为困难的一年。面对严峻复杂的国际国内经济形势，在省委、省政府和市委的坚强领导下，市政府带领全市人民戮力同心，共克时艰，全力以赴保增长、保民生、保稳定，完成了市十四届人大二次会议确定的各项任务。

　　预计全市地区生产总值实现4359亿元，比上年增长14.1%；全社会固定资产投资完成3676亿元，增长22.2%；社会消费品零售总额实现1778.6亿元，增长18.1%；实际利用外商直接投资54.1亿美元，增长2.3%；地方财政一般预算收入完成320.2亿元，增长10%；规模以上工业增加值实现2017.5亿元，增长19.1%。预计主要经济指标在全国副省级城市中继续位居前列。

　　（一）以保增长为首要任务，全力推动经济平稳较快发展

　　坚持立足当前、着眼长远，把保增长与调结构结合起来，努力增强经济发展后劲。

　　全面实施保增长各项政策措施。及时出台支持企业发展促进经济增长30条，筹集30亿元产业发展资金，减轻企业负担16.4亿元，加强生产要素调度，开展“进企业解难题、实施保增长工程”，有效扭转了经济增速下滑态势。出台促进开发区和城区加快发展的若干意见，激发活力，释放潜力。压缩行政开支，全力保证重点支出，发挥了财政对经济增长的促进作用。

　　努力扩大投资促进消费。坚持以项目建设带动投资增长，全年开工建设投资3000万元以上项目1849个。争取中央投资项目322个，总投资97.5亿元，争取地方债券15亿元，发行信托计划15亿元、企业债券12亿元，与中国建设银行等5家银行签订2700亿元贷款战略合作协议，金融机构本外币贷款增加1204亿元。推动“家电下乡”、“汽车摩托车下乡”和“汽车以旧换新”，进一步挖掘消费潜力。房地产开发投资完成1188.7亿元，增长17.6%；商品房销售面积达到1532.9万平方米，增长4.6%，充分发挥了拉动投资、带动消费的双重作用。

　　积极推动产业结构优化升级。加快建设具有国际竞争力的先进装备制造业基地，规模以上装备制造业增加值占全市比重达到48%。《沈阳铁西装备制造业聚集区产业发展规划》获国家批准，北方重工新基地、沈化集团50万吨CPP等重点项目竣工投产。现代建筑产业园全面启动，航高基地建设步伐加快。汽车产业扭转下滑局面，实现大幅回升。加快服务业集聚区建设，服务业增加值实现1948亿元，现代服务业比重达到52.4%。全国第一个金融外包服务中心在沈揭牌，金融要素市场建设取得突破性进展，金融商贸开发区正在成为全市第五大发展空间。泗水科技城、华强文化科技产业园等重大项目扎实推进，浑南动漫产业基地综合实力进入全国前列，保税物流中心封关运行，商务会展交易额突破1200亿元。大力发展县域经济，新增设施农业20万亩，农业生产战胜严重旱灾、再获丰收，于洪五金等8个县域产业集群达到百亿规模，县域地区生产总值增长16.7%，占全市比重达到39.8%。

　　进一步提升自主创新能力。全市高新技术产品增加值实现840亿元，增长22%。中科仪罗茨干泵等20个项目列入国家重大科技专项。成功并购9家海外科技型企业，引进海外研发团队50个。辉山农业科技园跻身国家级行列，我市成为“国家知识产权工作示范城市”。

　　（二）以创建“样板城”为契机，加快推进生态市建设

　　坚持资源节约和生态保护，推进城乡重大基础设施建设。城建投资完成360亿元，增长26.8%。

　　启动全国环境建设样板城创建工作。制定了五年建设总体目标，规划了100平方公里国家现代环保产业园，中钢北方金属资源加工等一批静脉产业项目成功签约。我市被联合国环境规划署列为全国唯一的生态城示范项目。

　　加强城乡基础设施建设。哈大客运专线、沈铁城际铁路等重点工程取得积极进展，沈抚城际铁路、沈康高速二期实现通车，地铁1号线全线轨通，1号线东延线、2号线北延线开工建设。94座危旧桥梁完成改造，沈北、五爱客运枢纽全面竣工。沈阳展览中心开工建设，奥体中心网球馆、游泳馆投入使用。启动大伙房水库输水配套工程，康平电厂并网发电。

　　着力改善城乡生态环境。建成22座辽河流域污水处理厂和一批村镇污水、垃圾处理设施，高标准实施浑河生态提升工程。植树1205万株，造林49万亩，完成500个中心村绿化建设。东陵区通过国家生态区验收，沈北新区、棋盘山开发区通过国家技术核查，和平区成为全省首个生态城区，30个乡镇通过国家环境优美乡镇验收。

　　深入推进节能减排。金山热电、沈西热电“上大压小”项目开工建设，新北热电等6家企业脱硫工程全面展开，沈海热电脱硫改造全面完成。全市单位地区生产总值能耗下降4.5%，化学需氧量、二氧化硫排放量分别减少3.2%和4.5%，提前完成“十一五”减排目标。

　　（三）以增强发展动力和活力为目的，不断深化改革开放

　　坚持用改革的办法解决前进中的问题，通过扩大开放谋求发展机遇，不断创新体制机制，努力提高对外开放水平。

　　加快重点领域和关键环节改革。完成商业城集团整体改制。新松机器人等5家企业成功上市。组建和完善基础产业集团等5家投融资平台，沈阳联合产权交易所获得金融企业省级国有产权交易机构资格，首家村镇银行和15家小额贷款公司正式开业，法库成为“全国农村金融产品和服务方式创新试点县”。出台促进非公有制经济和中小企业发展20条，沈北新区成为全国唯一的国际中小企业创业基地，非公有制经济增加值占全市地区生产总值比重提高到65.1%。开展农村土地承包经营权流转市场建设试点，政策性农业保险工作全面推进。积极稳妥实施水价和环保收费改革，圆满完成政府机构改革，医药卫生体制改革走在全国前列。

　　努力扩大对外开放。创新招商方式，优化引资结构，全年引进超千万美元外资项目64个，新增世界500强企业6家，引进投资500万元以上内资项目1809个，调入资金617.2亿元。成功引进日本精工、安川电机、油研工业等一批世界著名装备制造企业，世界最大的现代建筑及开发企业日本鹿岛建设进入我市。积极开展对外经贸合作，全市实现外贸出口35亿美元，新签对外工程承包与劳务合作合同额10.2亿美元，沈阳动力集团参与兴建美国第二大风能项目，并在美国投资建厂。成功举办制博会、“德中同行”等10余项大型活动，沈阳国际知名度和影响力显著提升。

　　加快沈阳经济区一体化进程。道路交通等基础设施建设扎实推进，旅游集散中心正式挂牌运行，沈抚连接带浑河南岸工业园区建设步伐加快。积极发挥沈阳经济区首位城市作用，推动国家新型工业化综合配套改革试验区申报工作取得重大进展。

（四）以保障改善民生为重点，全面加强社会建设

　　坚持把保障和改善民生作为政府工作的出发点和落脚点，克服财政减收增支的突出矛盾，完成民生工程投入226.6亿元，增长13.2%。

　　千方百计稳定和扩大就业。出台促进就业创业70条，推动以创业带动就业，加大就业援助力度，全市实名制就业25.1万人，城镇登记失业率控制在3.2%。10万名高校毕业生实现就业，困难家庭大学生全部安置工作。扩大集体合同覆盖面，加强农民工工资清欠和困难职工帮扶，促进了劳动关系和谐发展。

　　不断完善社会保障体系。启动新型农村社会养老保险试点和大学生参加居民医保工作，实施农民工医疗工伤综合保险，新型农村合作医疗参合率达到99.3%，人均筹资标准提高到150元以上。11类低收入居民及部分特殊群体待遇标准得到进一步提高。建设5个中心城区示范性区域养老中心，新建和改扩建12所农村区域性中心养老院。为2.2万户城市低收入住房困难家庭新建经济适用住房和发放货币补贴，为农村贫困户建房2250套。

　　切实提高城乡居民生活质量。城市居民人均可支配收入达到18560元，增长9.1%，农民人均纯收入实现9129元，增长14%。推进事业单位工资制度改革，在全省率先实施义务教育学校教师绩效工资制度，落实义务教育阶段教师津贴补贴政策。发放农资综合直补等政策性补贴。改造256个老旧小区、149条街巷路，新开和调整60条城市公交线路和农村客运班线，新建511公里农村公路，建成395处农村饮水安全工程。

　　大力发展社会事业。全面完成104所农村九年一贯制学校建设和质量再提升工程，完成20所普通高中标准化建设，6所万人规模职业技术学校全部投入使用。市四院、七院、奉天医院交付使用，市骨科医院和妇婴医院完成主体工程，儿童医院完成改扩建一期工程，新建和改扩建35所乡镇卫生院和14个社区卫生服务中心，甲型H1N1流感得到有效防控。文化事业蓬勃发展，8部文艺作品获得13个国家级奖项，我市荣获“全国文化体制改革先进地区”称号。沈阳体育健儿在全运会上取得12.5枚金牌的骄人战绩。

　　全面加强社会管理。积极探索信访工作新模式，加大调处力度，解决了4万多户居民无法办理房证、“荷兰村”历史遗留问题等一大批信访积案，全市信访稳定形势发生根本性好转，得到国家充分肯定，“沈阳经验”在全国推广。沈河区、大东区被评为全国和谐社会建设示范单位。96123“一号通”市民服务热线开通，铁西区数字化城管系统通过国家验收。加强食品药品等安全监管，有效防范重特大安全事故发生。深入开展社会治安综合整治行动，加大对各类严重刑事犯罪打击力度，国庆安保实现了“大事不出、小事也不出、万无一失”的庄严承诺，人民群众的安全感和满意度明显上升。

　　全面推进法制政府建设，依法行政能力和水平进一步提高，拥军优属、军民共建活动深入开展，民兵预备役工作不断加强，人口与计划生育、统计、监察、审计、参事、民族、宗教、侨务、广播、电视、新闻、出版、税务、工商行政管理、气象、防震减灾、人防、档案、修志、文史、红十字会、残疾人事业和老龄工作也都取得了新成绩。

　　各位代表，过去一年，面对国际金融危机的严重冲击，我市继续保持了经济社会平稳较快发展，成绩的取得极为不易。这是省委、省政府和市委正确领导的结果，是市人大、市政协大力支持的结果，是全市人民同舟共济、拼搏奋斗的结果。我代表市政府，向全市各族人民致以亲切问候和崇高敬意！向人大代表、政协委员，各民主党派、工商联、各人民团体和各界人士，向参与沈阳发展建设的中央和省在沈单位、驻沈部队指战员、武警官兵、港澳台同胞、海外侨胞及国际友人表示衷心感谢！

　　在充分肯定成绩的同时，我们必须清醒地看到，我市经济社会发展仍面临许多困难和问题。主要表现在：经济增长质量还不够高，部分城区财政收入增长乏力，收支矛盾比较突出；具有牵动力、影响力的重大项目还不够多，经济发展后劲还不太足；土地节约集约利用水平不高，土地综合收益较低；一些政府工作人员服务意识淡薄，软环境建设亟待加强；涉及群众切身利益的问题仍然较多，保障改善民生的任务还很繁重。对此，我们必须高度重视，切实加以解决。

　　二、2010年政府工作安排

　　今年是实施“十一五”规划的最后一年，也是全面完成“五大任务”、加快实现“三大目标”的关键之年，做好今年工作意义十分重大。我们要满怀发展激情，抢抓发展机遇，精心谋划，扎实工作，推动经济社会又好又快发展。

　　今年政府工作的总体要求是：认真贯彻党的十七大、十七届三中、四中全会和省委十届九次全会精神，深入贯彻落实科学发展观，按照市委十一届八次全会部署，紧紧围绕促进发展方式转变，着力调整优化经济结构，大力推进改革开放和自主创新，切实加强城市建设管理和生态环境建设，更加注重保障和改善民生，立足新起点，谋求新发展，实现新跨越，全面完成“十一五”规划确定的各项任务，为“十二五”规划启动实施奠定坚实基础。

　　全市经济社会发展主要预期目标是：地区生产总值增长14%，全社会固定资产投资增长20%，社会消费品零售总额增长16%，实际利用外商直接投资增长10%，地方财政一般预算收入增长11%，城市居民人均可支配收入和农民人均纯收入分别增长11%和13%，城镇登记失业率控制在4%以内。

　　（一）加大结构调整力度，积极促进经济平稳较快发展

　　全面落实国家重点产业调整振兴规划，把推进产业结构调整与做优重点发展空间结合起来，以增量调结构，在调整中扩增量，进一步提高经济增长质量和效益。

　　继续加大投资力度。强力推进投资3000万元以上项目建设，重点推进900个在建项目和1000个新开工项目，着力引进和培育一批重大项目，促进世界500强企业和中央直属大型企业落户沈阳，确保600个项目签约落地。优化投资结构，重点投向城乡重大基础设施和“三农”、民生、节能环保、自主创新、技术改造等领域。加大土地一级整理力度，进一步规范土地出让，努力提高土地综合收益。积极争取国家资金，鼓励社会及外商投资，加强资金筹集与项目投资的有效衔接。努力扩大消费需求，鼓励住房、汽车等大宗消费，促进教育、文化、旅游、家政、养老等服务消费，进一步开拓农村消费市场。

　　大力促进产业优化升级。推进信息化与工业化融合发展，努力扩大工业发展规模、提升工业发展质量，进一步提高装备制造业比重，全市规模以上工业增加值增长18%。紧紧抓住铁西装备制造业聚集区发展上升为国家战略的有利时机，加快建设机床、输变电、现代建筑、通用装备和汽车及零部件等5大千亿元产业，做大做强机床、沈鼓、远大、北方重工、三一重装等一批骨干企业，培育一批具有国际竞争力的世界级产品，积极发展工程成套和装备成套，加快装备制造业公共检测等服务平台建设，启动实施投资5.6亿欧元的华晨宝马扩能、投资15亿美元的米其林搬迁扩产等重大项目。加快发展现代建筑产业，整合优势资源，重点推进现代建筑产业园建设，努力打造现代建筑产业之都。加快航高基地建设，加强与中航集团、南航集团和庞巴迪公司的战略合作。积极推进汽车产业聚集区建设，扩充产业链，扩大品牌影响力，提高市场占有率。

　　切实发挥城区发展服务业实施主体作用，推动服务业特别是现代服务业大发展、快发展，全市服务业增加值增长13.5%，现代服务业比重达到55%。加快沈阳金融商贸开发区和金融街建设，大力引进国内外金融机构，积极发展沈阳钢材期货等金融要素市场，建设东北区域金融中心。加强国药物流等重点项目建设，引进培育一批第三方物流企业，推动保税物流中心升级为综合保税区。加快大中街、太原街、南塔、长江街等商贸集聚区改造升级，积极引进国内外一流的商贸服务企业和总经销商、总代理商。推进华强文化科技产业园等重大项目建设，发挥棋盘山国家文化产业示范区先导作用，做强文化旅游产业。大力发展楼宇经济和总部经济，积极引进和培育国际国内品牌展会。促进房地产业平稳健康发展，增加普通商品住房供给，鼓励居民自住和改善性购房，大力发展二手房市场和住房租赁市场。加大金廊建设力度，完善基础设施配套，加快在建项目建设，做好重点地块招商和在建项目、闲置楼宇的二次招商。

　　进一步做大做强县域经济，县域地区生产总值增长20%。推进法库辉山乳业、新民林浆纸等重大项目建设，培育于洪装备制造业配套工业园、苏家屯有色金属加工园等新型工业园区，形成一批新的百亿元产业集群。加强农田水利等基础设施建设，改善农业生产条件，提高农业综合生产能力，稳定粮食生产，保障主要农产品供给。突出抓好现代农业示范区建设，加快发展“一县一业”，新增设施农业10万亩、高效特色农业21万亩，新增投资1000万元以上农产品深加工项目80个。加强农产品产地市场和批发市场建设，深入推进“万村千乡”市场工程和“新网工程”。加强4个县城及胡台、近海、空港等新城规划建设，加快14个中心镇基础设施和公共设施建设，促进人口、资源、产业向重点城镇集中，形成城镇化与新农村建设良性互动的新局面。

　　立足我市现有基础和优势，大力发展新兴产业。高水平编制总体规划和专项规划，制定扶持政策，推动电子信息、新能源、新材料、生物制药、节能环保、民用航空等产业发展。加快康平碳纤维、沈北五洲龙混合动力客车等重大项目建设。积极推进泗水科技城建设，以现代生物技术为支撑，着力打造产业特色鲜明的科技园区。

　　推进自主创新和节能减排。加快实施国家重大科技专项，积极推进“双百工程”，支持国家级工程研究中心、工程实验室和企业技术中心建设，加强核能、纳米、物联网、新型动力设备等领域的技术研究与应用。加大企业技术改造力度，积极争取东北地区等老工业基地调整改造专项。加强产学研一体化合作，支持东北大学、沈阳工业大学等国家级大学科技园建设。积极申报国家创新型城市。大力推进节能减排，单位地区生产总值能耗下降4.5%，化学需氧量、二氧化硫排放量分别控制在6.6万吨和10.5万吨以内。大力发展清洁能源，推广使用高效节能产品，推进工业领域电机系统变频改造，实施沈煤公司等重点企业节能工程。推行清洁生产，推进排污集中控制，加快沈化、东药等高排放企业整体搬迁改造，淘汰沈阳热电、金山热电小发电机组，继续实施35吨以上燃煤锅炉脱硫工程。

　　大力推进沈阳经济区一体化发展。抓紧编制实施《沈阳经济区城际连接带发展规划》，推进基础设施、产业、新兴城镇的连接。加快城际交通、能源、环保等重大基础设施建设，大力推进通讯、市场、户籍管理等方面一体化。加快重要节点上的产业园区建设，明确功能定位，实现错位发展。采取同城化、珠链式、组团式发展模式，加快沈阳经济区城际连接带建设，围绕母城扩大城市规模。制定和完善各项政策措施，加强土地、资金、人才等要素保障。积极申报国家综合配套改革试验区，争取沈阳经济区发展上升为国家战略。

　　（二）加强基础设施建设和生态环境建设，切实提高城市宜居水平

　　抓住筹备第十二届全运会的有利时机，统筹规划建设基础设施，完善城市运行保障体系，提升城市功能，加强生态环境保护，全面推进环境样板城建设。

　　高水平制定城市发展规划。抓紧实施行政区划局部调整，合理配置空间资源，进一步做优发展空间。科学制定沈阳全域发展规划，完成《沈阳市城市总体规划》修编。完善重点产业空间规划，促进各行政区与功能区有效衔接。增强规划意识，切实维护规划的权威性和严肃性。

　　加大基础设施建设力度。启动第十二届全运会比赛场馆、运动员村、媒体公寓及相关配套服务设施建设。加快建设艺术中心、新世界会展中心等公共设施，建成沈阳展览中心。全面实施《沈阳市综合交通规划》，启动南北二干线等城区快速交通系统建设，改造和建设一批城市桥梁、市政道路、地下人行通道，规划建设一批停车场。加快综合交通客运枢纽、公交换乘枢纽和县级客运站建设。确保地铁1号线建成运营，2号线基本完成土建工程，加快1号线东延线和2号线北延线建设。加快沈铁城际铁路、京沈、沈丹客运专线及沈阳北客站等铁路客运专线枢纽站建设，积极推进新铁高速公路、沈本开发大道、沈抚2号公路、沈铁2号公路建设。加快大伙房水库输水配套工程建设。

　　进一步加强城市管理。健全城市管理长效机制，全面推进数字化城管。加大综合执法力度，开展市容环境和交通秩序综合整治，清理整顿街路市场，强化市政设施、园林绿化、户外广告管理。出台弃管小区管理办法，加强弃管小区管理。

　　努力改善生态环境。全面推进辽河、浑河、白塔堡河、南北运河综合整治，加大卧龙湖生态环境保护力度，实施蒲河生态建设三年规划，加强秀湖、丁香湖、珍珠湖等水系建设，打造沈阳北部生态廊道。新建一批污水处理厂和村镇污水处理设施，启动污泥无害化处置，推进中水回用、雨污分流、垃圾分类回收。实施规模化畜禽养殖企业污染治理。开展大规模绿化造林，实施荒山生态恢复、沈西北防风阻沙带工程，加快辽河水系绿洲、蒲河湿地森林景观带和县域工业园区生态绿化隔离带建设，实施500个中心村绿化，植树造林40万亩。

　　深入推进全国环境建设样板城创建工作。高标准制定总体规划和专项规划。加快国家现代环保产业园建设，大力发展静脉产业。启动国家级生态工业园区试点。发展绿色建筑和绿色交通，推进政府绿色采购，广泛开展绿色机关、社区、家庭创建活动。积极申报国家低碳经济试点城市。

　　（三）不断深化改革开放，进一步完善有利于科学发展的体制机制

　　加快推进重点领域和关键环节改革，不断拓展对外开放的广度和深度，进一步增强经济社会发展的动力和活力。

　　推进行政管理体制改革。完善大部门管理体制，深化行政审批制度改革，健全政府绩效评估指标体系。积极稳妥地推进事业单位分类改革。整合土地储备交易机构，依法严格实行土地集中统一管理。深化政务公开，加强信用政府建设，切实发挥政务诚信在“信用沈阳”建设中的引领示范作用。

　　加快医药卫生体制改革。继续扩大基本医疗保障覆盖面，提高保障水平。在政府举办的基层医疗卫生机构建立基本药物制度，努力实现零差率销售。逐步完善基层医疗卫生服务保障体系，推进基本和重大公共卫生服务项目建设。积极探索公立医院改革试点。

　　深化国有企业改革。积极引进战略投资者，鼓励各类投资主体参与国有企业改革重组。加快重点国有企业股份制改造，推动东药集团与红梅集团重组。支持重点骨干企业实行跨区域、跨行业、跨所有制战略重组，着力打造具有国际竞争力的大型企业集团。

　　切实抓好财政金融体制改革。推进政府预算体系建设，加强社保基金预算管理。优化财税结构，切实增强财政增收的稳定性和可持续性。强化政府性债务管理，控制债务规模，防范债务风险。拓展沈阳联合产权交易所功能，推动盛京银行上市。支持沈阳金融商贸开发区晋升国家级开发区。积极争取我市成为跨境贸易人民币结算试点城市。稳步推进资源性产品价格改革和环保收费改革。

　　深化农村综合改革。创新土地流转方式和途径，推进土地适度规模经营，继续深化集体林权制度配套改革。加快农村信用社、村镇银行、小额贷款公司等金融服务机构建设。支持农民专业合作社和产业带头人队伍发展壮大。推动供销合作社、农垦总公司改革发展。

　　大力支持非公有制经济和中小企业发展。支持民间资本进入基础设施、公用事业、金融服务和社会事业等领域。鼓励中小企业向“专精特优”方向发展，与汽车、机械装备等重点骨干企业协作配套，增强市场竞争力。落实专项扶持资金，完善创业融资服务体系，支持中小企业上市。

　　进一步扩大对外开放。努力改善投资环境，加大招商引资力度，优化利用外资结构，引进超千万美元外资项目50个，实际利用外商直接投资60亿美元；引进投资500万元以上内资项目1800个，调入资金700亿元。扩大高新技术产品、大型成套设备出口，大力发展服务贸易和服务外包，全市出口总额增长20%。支持企业“走出去”，开展境外资源合作开发、劳务合作、工程承包。精心办好制博会、东北亚发展论坛，加快沈阳桃仙国际枢纽港建设。

　　（四）大力发展社会事业，着力保障和改善民生

　　继续实施“为城乡群众办实事”工程，集中力量办一些涉及人民群众切身利益的大事实事，为人民群众谋求更多福祉。

　　加大就业促进力度。完善就业和再就业各项政策，促进创业带动就业，支持群团组织“牵手同行”就业创业联合行动，多渠道增加就业岗位，实名制就业25万人。做好高校毕业生、复员军人及城镇新增劳动力就业工作。加大零就业家庭等困难人员就业援助力度，加强农村劳动力转移就业培训，普惠制就业培训6万人。规范人力资源市场秩序和企业用工行为，进一步落实集体合同制度，维护劳动者合法权益。

　　增加城乡居民收入。完善职工薪酬制度和公务员津贴补贴政策，推进事业单位绩效工资制度改革。提高低收入居民及部分特殊群体待遇标准。落实涉农补贴政策，增加农民务农收益和务工收入。采取积极有效措施预防和解决农民工工资拖欠问题，确保农民工工资按时足额发放。

　　完善城乡社会保障体系。强力推进养老保险参保扩面，积极开展农村新型社会养老保险试点,加大事业单位养老保险制度改革力度。大力推进大学生参加城镇居民基本医疗保险、农民工参加医疗工伤综合保险，职工医疗保险和生育保险实行市级统筹，进一步提高新型农村合作医疗补助标准和参合率。完善低保户、低保边缘户救助制度，加强对困难职工的帮扶。大力发展社会福利和慈善事业，完善城乡养老服务体系，认真做好残疾人工作。切实解决城乡低收入家庭住房困难，加快非集中联片棚户区改造。解决农村人口饮水安全问题。

　　加快教育卫生文化体育事业发展。启动教育强区县创建活动，推进城乡义务教育高水平均衡发展，加强普通高中标准化、特色化建设，推进职业学校开展国际化合作，积极申报全国职业教育试点城市，加快构建终身教育体系，支持在沈高校发展。启动市精神卫生中心等改扩建工程，加快市一院改扩建进度，市儿童医院、骨科医院、妇婴医院新建和扩建工程全部完成并投入使用，推进4所县级中心医院建设，建成35所乡镇卫生院、14个社区卫生服务中心，做好甲型H1N1流感和重大传染病疫情的防治工作。建设沈阳博物馆、艺术大厦等文化设施，打造艺术精品。开展全民健身活动，办好第十一届省运会。

　　保持社会和谐稳定。进一步完善信访稳定工作“沈阳模式”，健全信访稳定工作风险评估机制，及时解决群众反映强烈的信访问题，积极预防和依法妥善处置群体性事件。提高应急管理工作水平，有效应对各类突发事件。完善市场供应保障体系，加大食品药品监督抽查力度，保障食品药品和公共安全。加强安全生产监督管理，坚决遏制重特大事故发生。加快构建社区服务体系，深入推进社会治安防控体系建设，依法严厉打击各类犯罪行为，增强人民群众安全感。加强民兵预备役工作，积极支持驻军建设，做好优抚安置工作，开创“双拥共建”新局面。

　　认真做好“十二五”规划编制工作，为我市经济社会又好又快发展提供科学指导。

　　各位代表，完成全年各项任务对政府自身建设提出了新的更高要求。各级政府要把软环境建设放在更加突出的位置，坚持依法行政，自觉接受人大依法监督和政协民主监督，支持人民团体发挥作用，努力提高科学民主决策水平，进一步加强廉政建设。各级政府工作人员特别是领导干部，要切实转变工作作风，增强服务意识，提高行政能力，加强廉洁自律，重实际、求实效、办实事，打造一支作风过硬的公务员队伍，让人民放心，让人民满意。

　　各位代表，全面振兴沈阳老工业基地，任务艰巨而繁重，使命神圣而光荣。让我们在省委、省政府和市委的正确领导下，深入贯彻落实科学发展观，咬定发展目标，只争朝夕，埋头苦干，为加快实现沈阳老工业基地全面振兴而努力奋斗!
